# Portal-Mesenteric Vein Resection in Borderline Pancreatic Cancer; 33 Month-Survival in Patients with Good Performance Status

**DOI:** 10.1089/pancan.2019.0013

**Published:** 2019-09-26

**Authors:** Gregory G. Tsiotos, Nikiforos Ballian, Theodoros Michelakos, Fotios Milas, Panoraia Ziogou, Dimitrios Papaioannou, Charitini Salla, Ilias Athanasiadis, Evangelia Razis, Flora Stavridi, Maria Psomas

**Affiliations:** ^1^ Department of Surgery, Mitera-Hygeia Hospitals, Marousi, Greece.; ^2^ Department of Pathology, Mitera-Hygeia Hospitals, Marousi, Greece.; ^3^ Department of Cytology, Mitera-Hygeia Hospitals, Marousi, Greece.; ^4^ Department of Medical Oncology, Mitera-Hygeia Hospitals, Marousi, Greece.; ^5^ Department of Anesthesiology, Mitera-Hygeia Hospitals, Marousi, Greece.

**Keywords:** borderline pancreatic cancer, locally advanced pancreatic cancer, mesenteric vein resection, pancreaticoduodenectomy, portal vein resection

## Abstract

**Background:** Patients with pancreatic cancer (PC), which is not upfront resectable, but borderline, involving major peripancreatic vessels, have not been generally considered for surgery, considering that resection in such a setting may be futile.

**Materials and Methods:** Retrospective analysis of prospectively collected data on patients with borderline pancreatic adenocarcinoma undergoing pancreatectomy en-block with portal and/or superior mesenteric vein resection in a tertiary referral center in Greece between January 2012 and February 2017. Follow-up was complete up to January 2018.

**Results:** Twenty-four patients were included. Neoadjuvant therapy (NAT) was administered to only 38%, but more commonly in the second half of the group (58% vs. 17%, *p* = 0.035). It was associated with smaller tumor size (median: 2.5 vs. 4.2 cm, *p* < 0.001), fewer positive lymph nodes (LNs) in the resected specimen (median: 2 vs. 5, *p* = 0.04), and higher likelihood of adjuvant therapy (78% vs. 40%, *p* = 0.01), but not with survival. Resection was extensive: a median of 26 LNs were retrieved, R0 resection rate (≥1 mm) was 79%, and median length of vein segments was 4 cm, requiring interposition grafts in 58% (mostly polytetrafluoroethylene). Median intensive care unit stay was 0 days and length of hospital stay was 9 days. Post-operative mortality was 12.5%. Median overall survival was 24 months. Eastern Cooperative Oncology Group (ECOG) status was significantly associated with survival (*p* < 0.001) with ECOG-0: 33 months, ECOG-1: 12 months, and ECOG-2: 6 months.

**Conclusion:** This first Greek national series of portomesenteric vein resection in borderline PC demonstrates that it results to 2 years of median survival, extending to 33 months in patients with good performance status, especially if NAT is uniformly administered.

## Introduction

Management of pancreatic cancer (PC) with curative intent has made significant progress,^1^ especially after the recognition that more patients with previously considered unresectable disease could be offered a curative operation following neoadjuvant therapy (NAT—chemotherapy/chemoradiation).^2–5^ Surgical technique has also advanced, so that tumors involving major peripancreatic vasculature, once considered unresectable, are now safely removed in association with these major vessels in specialized centers.^3,6–8^ Currently, “borderline resectable” tumors (National Comprehensive Cancer Network [NCCN] criteria) are considered *technically* upfront resectable, but resection leads to improved outcome when preceded by NAT.^9^

However, there is still pessimism in the medical oncology community for patients with PC who are referred for chemotherapy without a previous pancreatectomy. Such patients, with locally advanced, but not metastatic disease, tend to be managed with only palliative intent and often not channeled to a pancreatic surgeon for a possible resection. In this context, our team, with a dedicated interest in pancreatic surgery, started performing major vascular resections in patients with borderline resectable PC. Our aims in this study were to analyze our initial experience with these patients, study details on venous resection, investigate time trends in administration of NAT and its possible correlations with tumor characteristics, and assess long-term results.

## Materials and Methods

Data on all patients who underwent pancreas resection associated with some portion of the superior mesenteric and/or portal vein, because of involvement by the tumor, at our division (>30 pancreas operations/year) between January 2012 and February 2017, were prospectively collected and retrospectively analyzed. Only patients with pancreatic ductal carcinoma were included. Age, sex, Eastern Cooperative Oncology Group (ECOG) performance status, type of pancreatectomy, duration of intensive care unit (ICU) and hospital stay, blood transfusions, administration of NAT and/or adjuvant therapy, and type of vein resection and reconstruction were recorded. Tumor size, number of lymph nodes (LNs), TNM stage (American Joint Committee on Cancer), and vein wall histologic involvement were noted. R0 resection was defined as negative margins of at least 1 mm.^10^ Post-operative complications, need for reoperation, and 90-day mortality were recorded. Follow-up was complete to patients' death, or up to January 2018. Data collection and the study were approved by our Institutional Review Board.

All patients were staged pre-operatively by computerized tomography. A PC was deemed borderline resectable when it met the NCCN criteria^11^ of venous involvement by the tumor allowing safe resection and replacement, short segment encasement or direct abutment of the hepatic artery without extension to the celiac artery, and abutment of the superior mesenteric artery not exceeding 180 ° of its circumference.

**FIG. 1.**
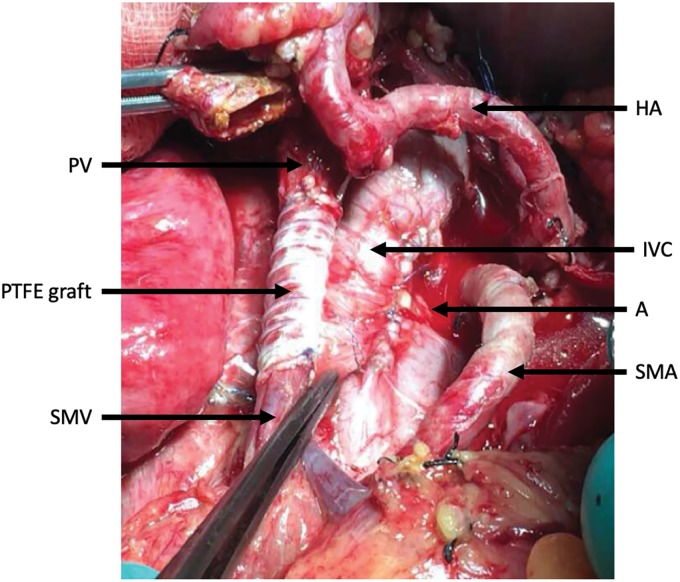
Complete skeletonization of the SMV from its first tributaries deep within the mesentery, PV up to the liver hilum, and HA and SMA from their take off, the anterior wall of the A and the IVC. PTFE graft is placed at the resected portion of the SMV. A, aorta; HA, hepatic artery; IVC, inferior vena cava; PTFE, polytetrafluoroethylene; PV, portal vein; SMA, superior mesenteric arteries; SMV, superior mesenteric vein.

Our operative technique includes complete skeletonization of the portal vein, superior mesenteric vein, and hepatic artery. In patients with tumors of the pancreatic body, complete skeletonization of the celiac trunk is also performed (Fig. 1A, B). LN dissection involves all standard peripancreatic LN beds. In all patients, venous resection was planned pre-operatively and was not a result of an inadvertent intraoperative event. When venous involvement with tumor was limited, a tangential excision was performed along the vein's longitudinal axis and was repaired with a transverse suture line. When a circumferential portion of the vein had to be resected, a primary end-to-end anastomosis was performed for a venous gap <3 cm, whereas an interposition prosthetic (polytetrafluoroethylene or PTFE) graft was used when >3 cm. We preferred prosthetic over venous interposition grafts, since these are readily available, limiting the operative time for native vein harvesting. Due to the extent of the disease, we proceeded more liberally to total pancreatectomy when appropriate. Patency of all prosthetic grafts was examined with ultrasonography 2 months post-operatively.

Continuous data are given as median and interquartile range (IQR), whereas categorical data are expressed as frequencies and percentages. Comparison of categorical variables among groups was performed using Fisher's exact test. Comparison of continuous variables between or within groups was performed using the Mann–Whitney U test. Given that our dataset included both patients who had or had not received NAT, and to homogenize the data, follow-up duration and overall survival (OS) were calculated from the time of diagnosis. Follow-up duration was calculated from the time of diagnosis to the time of death or last follow-up taking into account both dead and censored cases. Survival was calculated from the time of diagnosis to the time of death (event) or last follow-up (censored). Survival curves were plotted using the Kaplan–Meier method. Differences in OS between groups were analyzed by the log-rank test. Multivariate survival analyses were performed using the backward conditional Cox regression method. *p*-Value <0.05 was considered statistically significant. All tests used were two tailed. Statistical analysis was performed with the IBM SPSS Statistics software for Windows, version 20.0 (IBM Corp., Armonk, NY).

## Results

### Demographics

During the study period, 24 (*n* = 24) patients (17 males, 7 females) underwent pancreatectomy with resection of some part of the superior mesenteric vein and/or portal vein. Median age was 66 years (IQR: 60–72 years). ECOG performance status was graded 0, 1, and 2 in 11 (46%), 10 (42%), and 3 (12%) patients, respectively. The tumor was located at the body of the pancreas (seven patients, or 29%), uncinate process (seven patients, or 29%), neck (six patients, or 25%), and head (four patients, or 17%).

### Perioperative data

Total pancreatectomy (16 patients, or 67%) was the most common resection, followed by distal pancreatectomy (5 patients, or 21%), and Whipple operation (3 patients, or 12%). Total pancreatectomy was performed in all six patients with neck tumors, in two patients with large body tumors extending to the neck, in one patient with head tumor, and in three patients with uncinate tumors because of pre-existing insulin-dependent diabetes mellitus. The remaining four patients with uncinate tumors underwent total pancreatectomy because these tumors extended anteriorly toward the neck, involving the tissue between the superior mesenteric vein and artery. Portomesenteric venous resection was tangential in 5 patients (21%) and circumferential in 19 patients (79%). When circumferential, the median length of vein resected was 4.0 cm (IQR: 2.0–5.0 cm). Vein reconstruction was horizontal closure of the longitudinal tangential vein wall excision in 5 patients (21%), end-to-end anastomosis in 5 patients (21%), saphenous vein interposition graft in 1 patient (4%), and PTFE interposition graft in 13 patients (54%; Table 1).

**Table 1.** Clinicopathologic Characteristics of 24 Patients Who Underwent Pancreatic Resection with Vascular Resection for a Borderline Resectable Pancreatic Cancer

Characteristics	*n* (%)
Gender	
Female	7 (29)
Male	17 (71)
Age, years, median (IQR)	66 (60–72)
ECOG	
0	11 (46)
1	10 (42)
2	3 (12)
Location	
Body	7 (29)
Head	4 (17)
Neck	6 (25)
Uncinate	7 (29)
Neoadjuvant chemotherapy	
No	15 (63)
Yes	9 (38)
Operation	
Distal	5 (21)
Total	16 (67)
Whipple	3 (12)
Venous reconstruction type	
Primary	5 (21)
PTFE	13 (54)
Splenic vein	1 (4)
Tangential	5 (21)
Length resected (cm), median (IQR)	4 (2–5)
ICU stay	
No	16 (67)
Yes	8 (33)
ICU stay (days), median (IQR)	0 (0–1)
Transfused pRBC units, median (IQR)	2 (2–3)
Post-operative hospital LOS (days), median (IQR)	9 (7–17)
Adjuvant chemotherapy	
No	8 (33)
Yes	13 (54)
Tumor size (cm), median (IQR)	3.8 (2.9–5.5)
T	
T1	3 (13)
T2	3 (13)
T3	18 (75)
N	
N0	2 (8)
N1	22 (92)
Resection	
R0	19 (79)
R1	4 (17)
R2	1 (4)
Total LNs, median (IQR)	26 (20–33)
Positive LNs, median (IQR)	3 (2–6)
LN ratio (%), median (IQR)	9.5 (5.3–22.4)
Vein infiltration	
No	6 (25)
Yes	18 (75)
Complications	
No	16 (67)
Yes	8 (33)
Reoperation	
No	17 (81)
Yes	4 (19)

ICU, intensive care unit; LOS, length of stay; LN, lymph node.

Most patients (16, or 67%) did not require ICU stay (median ICU stay: 0 days, IQR: 0–1 days). The median number of units of packed red blood cells transfused perioperatively was 2 (IQR: 2–3). Length of hospital stay ranged from 5 to 30 days (median: 9 days [IQR: 7–17]).

Adjuvant chemotherapy (Gemcitabine alone) was administered to 13 of the 21 patients who were discharged from the hospital (62%). The remaining eight patients (38%) did not receive chemotherapy because five chose so, or because three were unfit.

### Neoadjuvant chemotherapy

No patient received chemoradiation. Nine (38%) received neoadjuvant chemotherapy: 6 Gemcitabine—Abraxane^®^, 2 FOLFIRINOX, and 1 both regimens. NAT was significantly more common in the second half of the group compared to the first half: 7 of the last 12 patients (58%), but only 2 of the first 12 patients (17%; *p* = 0.035). NAT led to significantly smaller tumor size (median: 2.5 vs. 4.2 cm, *p* < 0.001) and fewer positive LNs (median: 2 vs. 5, *p* = 0.04). Also, patients who received it were significantly more likely to continue with adjuvant therapy post-operatively (78% vs. 40%, *p* = 0.01). However, it did not correlate with total number of LNs, LN ratio, type or length of vascular resection, ECOG status, or survival.

### Pathologic findings

All patients had PC. The median tumor size was 3.8 cm (IQR: 2.9–5.5 cm). Six patients had tumors <3 cm and 5 of those had received NAT. The median number of peripancreatic LNs harvested was 26 (IQR: 20–33). A median of three LNs (IQR: 2–6) was infiltrated with cancer, for a median LN ratio of 9.5% (IQR: 5.3–22.4%). The only two patients with <15 LNs identified, had received NAT. The resected veins proved to be histologically infiltrated in most patients (18, or 75%), whereas in the remaining 6 patients (25%), the vessel wall was densely adherent to, but not infiltrated by, cancer. Of note, four of the latter six patients (67%) had undergone NAT. Indeed, vein wall infiltration was less frequent among patients after NAT (56% vs. 87%, *p* = 0.15). R0 resection (1 mm margin) was achieved in 19 patients (79%), R1 in 4 patients (17%), and R2 in 1 patient (4%).

### Morbidity and mortality

Prosthetic graft patency rate on 2-month post-operative ultrasound examination was 100%. In addition, no signs of infection, thrombosis, or anastomotic breakdown were encountered in any patient during follow-up. Eight patients (33%) developed at least one major complication: post-operative hemorrhage, wound dehiscence, grade II pancreatic fistula, pulmonary embolism, transverse colon necrosis, gastric staple line leak, and hepatic artery spasm with intrahepatic cholestasis and liver failure.

Four patients (17%) required reoperation and one patient (4%) a major intervention. Those with post-operative hemorrhage, wound dehiscence, and gastric leak, and one with colon necrosis were reoperated and did well eventually. The patient with hepatic artery spasm (diagnosed after severe metabolic acidosis, Doppler ultrasonography, and angiography) was subjected to emergency hepatic artery stent placement with resolution of the arterial stenosis from the origin of the artery to its bifurcation.

Three patients (12.5%) died 2, 5, and 30 days post-operatively. One patient (ECOG: 2) with significant intraoperative bleeding developed gut necrosis (the only one with R2 resection), one morbidly obese patient (body mass index of 47.3 kg/m^2^) developed massive pulmonary embolism, and one patient with hepatic artery spasm and emergency stent placement developed rapidly progressing intrahepatic cholestasis and died of liver failure.

### Survival analysis

The median follow-up duration from the time of diagnosis was 15 months (IQR: 9–29). Seven patients (33%) developed liver metastases and five patients (24%) peritoneal carcinomatosis. The median OS from the time of diagnosis was 15 months (IQR: 9–33; Fig. 2A). When the three patients who died within 30 days of the procedure were excluded from the survival analysis, median OS was 24 months (IQR: 12–34; Fig. 2B). The two patients with ECOG: 2 were operated very early in the study period. They survived only 4 and 6 months from diagnosis, thus skewing OS. Both died of carcinomatosis. Among the seven patients who were still alive at the end of the follow-up period, four patients (57%) had undergone NAT and only two patients have evidence of recurrent disease to date. Higher ECOG status was significantly associated with shorter survival on univariate analysis (median OS from time of diagnosis [IQR]: ECOG-0: 33 m [24-n/a], ECOG-1: 12 m [9–15], and ECOG-2: 6 m [4–6], *p* < 0.001; Fig. 3).

**FIG. 2.**
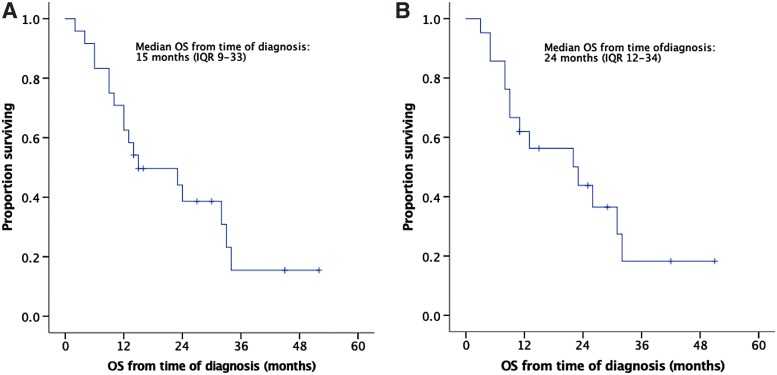
Kaplan–Meier OS curve of 24 patients who underwent pancreatic resection with vascular resection for a borderline resectable pancreatic cancer. OS is calculated from the time of diagnosis to death (event) or last follow-up (censored). **(A)** Complete cohort (*n* = 24). **(B)** Patients who died within 30 days of the procedure were excluded from this analysis (*n* = 21). IQR, interquartile range; OS, overall survival.

**FIG. 3.**
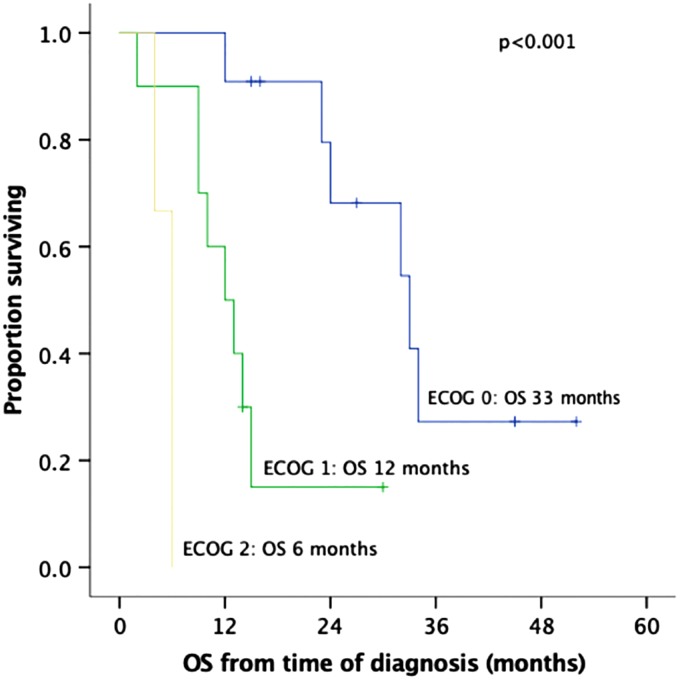
Kaplan–Meier OS curves of 24 patients who underwent pancreatic resection with vascular resection for a borderline resectable pancreatic adenocarcinoma, by ECOG category. OS is calculated from the time of diagnosis to death (event) or last follow-up (censored). ECOG, Eastern Cooperative Oncology Group.

## Discussion

Resection of the portomesenteric venous axis involved by PC has become common in pancreas referral centers.^3,5–8,12,13^ Two metanalyses^6,7^ proved that portal/mesenteric vein resection is safe in experienced hands (mortality 3–5%). It is also associated with similar survival to that of patients undergoing pancreatectomy with no involvement of this major vein.^3,4,6^ The routine addition of neoadjuvant chemotherapy with or without chemoradiotherapy has led to even better results,^4,14,15^ so that NAT followed by surgery in borderline PC belongs to the guidelines of the International Study Group of Pancreatic Surgery^9^ and is recommended by experts.^16,17^ Furthermore, with NAT and advanced surgical experience, 30–60% of patients with locally advanced disease (i.e., unresectable at diagnosis) undergo a curative resection.^2,4,5,18^

Despite this promising reality, pessimism still exists in parts of the medical and surgical communities in Greece: apart from patients with early, upfront resectable PC, almost all patients with borderline, or locally advanced (but not metastatic) PC are not considered for a possible resection, and chemotherapy is generally offered as the sole treatment modality. Such was the context in which we began operating on patients with borderline PC. Our team has a long dedication to pancreatic surgery and performs >30 pancreas resections annually. In this study, our experience with the first 24 patients is analyzed, management trends within these 5 years are explored, and long-term outcomes are reported.

To the best of our knowledge, this is the first series of patients with borderline resectable PC who underwent portomesenteric venous resection reported from Greece. In this nonselected group, NAT was administered mostly in the second half and resulted in smaller tumor size and fewer infiltrated LNs. Extensive oncologic resections were performed with a median of 26 LNs retrieved, 79% R0 resections (≥1 mm), and long vein segments resected (median 4 cm), with most (58%) requiring interposition grafts. PTFE grafts were not associated with long-term morbidity attributed to possible occlusion in follow-up. Median OS was 24 months, but it was significantly impacted by the two ECOG: 2 patients who lived for only 4 and 6 months. These early results appear very promising (given the inherent limitations of our group), but the post-operative mortality of 12.5% was very high.

The extent of pertinent LN dissection has now been standardized for a Whipple operation.^19,20^ Fifteen resected LNs are considered oncologically enough, but it has been suggested that 20 was the optimal number, especially in chemotherapy-naive patients.^21^ In our specimens, a median of 26 LNs were retrieved, which alludes to the extent of peripancreatic lymphoid tissue clearance and compares favorably to the number of examined LNs reported by most established pancreatic centers.^22^

The extent of our resections is also reflected in the 79% R0 resection rate (margin ≥1 mm), which compares favorably to the 55–96% “negative microscopic margin” rate reported by others,^2,23–26^ especially when considering that all patients in these studies had undergone NAT (vs. only 38% in ours) and margins <1 mm were considered negative (R0). Two thirds of our patients had to undergo total pancreatectomy, given the extent of their tumors and the absence of “downstaging” in most, since 63% had not received NAT. However, in this era of more extensive pancreatic surgery, total pancreatectomy has indeed become more frequent.^27^

Probably, the lack of NAT in most of our patients (63%) and the larger tumor size thereof (median 3.8 cm) were also associated with the rather long (median 4 cm) segments of portomesenteric vein resected. It was for this reason that 58% of our patients needed an interposition graft, as opposed to 21% each with primary end-to-end anastomosis or tangential repair. In contrast, in the Mayo Clinic experience,^28^ out of 89 patients, only 16% had vein segments long enough removed to necessitate a graft. Similarly, in the Heidelberg experience,^13^ in 82% of 110 patients, a tangential, or primary venous repair sufficed, whereas a graft was utilized in only 18%. Others have reported interposition grafts in 33% of 43 patients^29^ and 45% of 76 patients.^30^

Vein wall infiltration was histologically present in 75% of our patients, similar to 51–93%^12^ in 241 patients with portal vein resection who had not undergone NAT. Others have reported pathologically proven vein wall invasion by cancer cells in 51%,^30^ 77%,^31^ and 78%^13^ of patients. Because of the notorious lack of correlation between radiographic, operative, and pathologic findings after NAT,^12,18,24,32,33^ the strategy should be to proceed with an attempt at resection based on the significant decrease of CA 19-9,^23^ even if the tumor is radiographically “stable.” In our group, patients who did not receive NAT were more likely to have their resected vein histologically infiltrated (13 of 18 patients, or 72%), compared to those who received (2 of 6 patients, or 33%).

After feasibility and safety of portal vein resection for a curative pancreatectomy were confirmed in the 90s,^34,35^ overall median survival with upfront surgery (without any pre-operative treatment modality) ranged from 15 months^31^ to 22 months,^35^ and 23.4 months.^36^ With initiation of neoadjuvant chemotherapy, median survival after pancreatectomy in borderline PC has been reported ∼2 years: 23 months,^37–39^ 24 months,^40^ and 26 months.^25,41,42^ In centers of excellence and highly selected patients, median survival has now reached 3 years: 33 months,^24^ 35 months,^43^ 38 months,^4^ or 40 months.^44^ Our median OS of 24 months (33 months for patients with ECOG: 0) compares favorably to the literature, since only 38% of our group received NAT. In addition, our two patients with ECOG: 2, who were operated very early in our experience and survived for only 4 and 6 months due to early disease progression and carcinomatosis, skew the survival curve considerably. Performance status ≥2 has been recognized as an independent negative prognostic factor for survival after pancreatectomy,^45,46^ and is now considered a contraindication for surgery.^47^ At the same time, pre-habilitation may improve patients' physical strength and may increase the subgroup of ECOG: 0 patients who undergo resection leading to longer survival.^48^

Consensus has been reached that NAT is now an absolute pre-requisite in patients with borderline PC before resection is contemplated.^9,15,47^ Although in the first half of our study, utilization of NAT was scarce, this changed significantly in the second half (58% vs. 17%, *p* = 0.035), indicating the wider recognition in the medical and surgical oncology community that chemotherapy in patients with PC, which is not early and upfront resectable, but borderline, may not just be of palliative nature, but neoadjuvant with the goal to eventually proceed with resection. In our experience, NAT was significantly associated with smaller tumor size, fewer positive LNs, and higher likelihood for post-operative adjuvant therapy, which may allude to a higher level of commitment to thorough management in the neoadjuvant subgroup, by both patients and their oncologists. On the contrary, it proved not related to survival, but we believe that it was the small size of our group, its inhomogeneity, and the lack of standardized treatment that probably contributed to this finding. Similarly, NAT was not associated with the total number of LNs found in the specimen, performance status at operation, or the type and length of vein resection.

## Conclusion

In summary, the initial experience from Greece with pancreatectomy and en-block portomesenteric venous resection for borderline PC comprised a nonhomogeneous group of patients, most of whom did not receive NAT (especially the first half), who underwent extensive dissections (reflected in the high number of LNs, high R0 resection rate, long vein segments resected), did not need ICU admission, required minimal blood transfusions, and had a median OS of 24 months from diagnosis (33 months with ECOG: 0), which was very significantly lower in patients with ECOG ≥1. Although we need to diminish post-operative mortality, these initial results, which corroborate current literature, show that survival in patients with borderline PC can indeed be prolonged after extensive resections, including peripancreatic vasculature compared to only palliative care, and may serve as a springboard for a substantial increase of borderline and locally advanced PC patients with good performance status to undergo modern neoadjuvant protocols with the goal of curative resection and further survival improvement.

## Abbreviations Used

A: aorta
ECOG: Eastern Cooperative Oncology Group
HA: hepatic artery
ICU: intensive care unit
IQR: interquartile range
IVC: inferior vena cava
LN: lymph node
LOS: length of stay
NAT: neoadjuvant therapy
NCCN: National Comprehensive Cancer Network
OS: overall survival
PC: pancreatic cancer
PTFE: polytetrafluoroethylene
PV: portal vein
SMA: superior mesenteric arteries
SMV: superior mesenteric vein
